# Electroplating and magnetostructural characterization of multisegmented Co_54_Ni_46_/Co_85_Ni_15_ nanowires from single electrochemical bath in anodic alumina templates

**DOI:** 10.1186/1556-276X-8-263

**Published:** 2013-06-04

**Authors:** Victor M Prida, Javier García, Lucia Iglesias, Victor Vega, Detlef Görlitz, Kornelius Nielsch, Enrique Díaz Barriga-Castro, Raquel Mendoza-Reséndez, Arturo Ponce, Carlos Luna

**Affiliations:** 1 Departamento de Física, Universidad de Oviedo, Calvo Sotelo s/n, Oviedo 33007, Spain; 2 Institute of Applied Physics, University of Hamburg, Jungiusstraβe 11, Hamburg 20355, Germany; 3 Centro de Investigación de Ciencias Físico-Matemáticas/Facultad de Ciencias Físico-Matemáticas, Universidad Autónoma de Nuevo León, Monterrey, Nuevo Leon 66450, Mexico; 4 Facultad de Ingeniería Mecánica y Eléctrica, Universidad Autónoma de Nuevo León, San Nicolás de los Garza, Monterrey, Nuevo Leon 66450, Mexico; 5 Department of Physics and Astronomy, University of Texas at San Antonio, One UTSA Circle, San Antonio, TX 78249, USA

**Keywords:** Nanoporous alumina templates, Electrodeposition, Multisegmented nanowires, 61.46.-w, 68.37.Ma, 75.75.-c

## Abstract

Highly hexagonally ordered hard anodic aluminum oxide membranes, which have been modified by a thin cover layer of SiO_2_ deposited by atomic layer deposition method, were used as templates for the synthesis of electrodeposited magnetic Co-Ni nanowire arrays having diameters of around 180 to 200 nm and made of tens of segments with alternating compositions of Co_54_Ni_46_ and Co_85_Ni_15_. Each Co-Ni single segment has a mean length of around 290 nm for the Co_54_Ni_46_ alloy, whereas the length of the Co_85_Ni_15_ segments was around 430 nm. The composition and crystalline structure of each Co-Ni nanowire segment were determined by transmission electron microscopy and selected area electron diffraction techniques. The employed single-bath electrochemical nanowire growth method allows for tuning both the composition and crystalline structure of each individual Co-Ni segment. The room temperature magnetic behavior of the multisegmented Co-Ni nanowire arrays is also studied and correlated with their structural and morphological properties.

## Background

Research on nanostructures is motivated by the observation that material properties can abruptly change when scaling down the material size to nanoscale from its bulk counterpart mainly due to the enhanced surface-to-volume ratio of nanomaterials [1]. Several techniques have been reported for the synthesis of materials at nanoscale [2,3], but among these, the template-based method is a very simple and facile approach for obtaining dense metallic arrays with different geometries considered, such as planar and cylindrical nanostructures [4]. Chemical template-based methods combined with high-yield electrochemical deposition techniques have been recently employed to synthesize ordered arrays of magnetic nanowires and nanotubes [5,6]. The synthesis of nanostructured materials by means of electrochemical deposition into the nanopores of anodic aluminum oxide (AAO) membranes has attracted during the last decades a huge scientific interest due to the outstanding features exhibited by these templates such as low cost, large self-ordering degree of nanopores, high reproducibility, and precise control over their morphological characteristics [7]. These fabrication techniques based on combined bottom-up strategies allow fabricating magnetic nanoentities by electrochemically filling the AAO pores, and the amount of electrodeposited material can be easily controlled through the charge recorded during the nanowire growth. This makes possible the preparation of highly ordered nanostructures with specific dimensions and properties [8,9]. The peculiar characteristics of hard anodic aluminum oxide (H-AAO) membranes, mainly the low processing time, large interpore distances, and a broad window of self-ordering conditions, have demonstrated at the same time to be advantageous for their use as templates in the fabrication of highly ordered nanowire arrays [10]. The high nanoporous oxide growth rate achieved by means of hard anodization (HA) method (about 50 μm/h, 20 times faster than the standard mild anodization), together with the fast development of a hexagonal highly ordered nanoporous arrangement, allows us to produce H-AAO membranes with reproducible geometrical parameters in a few hours by only performing a single anodization step [11].

Increasing interest has been focused on the study of ferromagnetic/non-magnetic heterogeneous nanowire arrays [12,13], while only few works are devoted to heterogeneous ferromagnetic binary and segmented (barcode) nanowires [14,15]. Co-Ni alloy nanowires are outstanding magnetic materials that can exhibit both either a soft or hard magnetic behavior depending on the Co/Ni ratio in the alloy [16-18]. The combination of low magnetocrystalline anisotropy of face-centered cubic (fcc) Ni and high magnetocrystalline anisotropy of hexagonal close-packed (hcp) Co, together with the high solubility of Co atoms in the crystalline lattice of Ni and vice versa for a wide range of relative concentrations [18], allows for the design of a material composition with tunable magnetic properties. The effective magnetic anisotropy energy is determined by the competition between the shape and magnetocrystalline anisotropies, together with the magnetostatic dipolar interactions among nanowires, being possible to tune the easy magnetization direction of the system between the longitudinal and perpendicular directions with respect to the nanowire axis [19,20]. Additionally, the study on multisegmented magnetic nanowires, comprising alternate single segments of soft and hard magnetic materials with well-controlled thicknesses and separated by non-magnetic interspacers, has recently drawn the interest of the scientific community due to the interesting magnetization reversal processes that take place in these nanostructured materials that may allow for the design of multistable magnetic systems that are capable of storing several bits of information in a single nanowire [21]. Consequently, the design and fabrication of multisegmented magnetic nanowire arrays with an accurate control of the crystalline structure and magnetocrystalline anisotropy of each nanowire segment plays a key role in the design of nanostructured magnetic materials with a required magnetic behavior for tailoring the magnetic and magnetotransport performance of nanostructured systems and devices [22].

In the present work, highly hexagonally ordered H-AAO membranes, which have been modified by a thin cover layer of SiO_2_ deposited by atomic layer deposition (ALD) method, were used as templates for the synthesis of electrodeposited multisegmented Co_54_Ni_46_/Co_85_Ni_15_ nanowire arrays with a diameter ranging between 180 and 200 nm and the length of each individual Co-Ni segment depending on its particular composition (around 290 nm for the Co_54_Ni_46_ segments, while around 430 nm for the Co_85_Ni_15_ ones). The optimum synthesis conditions for obtaining such multisegmented nanowires were established by carefully studying the electroplating of homogeneous Co-Ni alloy nanowire arrays grown at several electrochemical deposition potentials in order to determine the deposition rate and chemical composition of the deposits grown at each electrodeposition potential.

The composition and crystalline structure of each segment of the Co_54_Ni_46_/Co_85_Ni_15_ nanowires were determined by transmission electron microscopy (TEM), energy dispersive X-ray spectroscopy (EDS), and selected area electron diffraction (SAED) techniques. The results indicate that our electrochemical growth method allows for tuning both the composition and crystalline structure of each individual Co-Ni segment deposited from a single electrolyte. The room temperature (RT) magnetic behavior of the multisegmented Co-Ni nanowire arrays has been also studied and correlated with their structural and morphological properties.

## Methods

High-purity aluminum foils (Al 99.999%, Goodfellow, Coraopolis, PA, USA) were firstly cleaned by means of ultrasonication in isopropanol and ethanol for 5 min. Afterwards, the Al foils were placed into the anodization cell and electropolished up to a mirror-like finishing in a vigorously stirred mixture of perchloric acid and ethanol (25:75 vol.%) at 5°C, with an applied voltage of 20 V measured versus a Pt counter electrode. The Al substrates were then pre-anodized under mild anodization conditions at 80 V for 10 min in a 0.3 M oxalic acid aqueous solution containing 5 vol.% of ethanol at a temperature between 0°C and 3°C. Afterwards, the anodization voltage was increased at 0.08 V s^−1^ to reach potentiostatic conditions in the HA process, which was carried out at 140 V for 1.5 h. After the HA process, H-AAO membranes were released from the unoxidized Al substrate, which was removed by wet chemical etching in a CuCl_2_/HCl aqueous solution, and the membranes were subsequently immersed for 2.5 h in 5 wt.% H_3_PO_4_ at 30°C in order to remove the alumina barrier layer at the bottom of the pores, also increasing the pore size of the H-AAO membranes. This last chemical etching step also results in a complete dissolution of the protective mild anodization AAO layer on the top of the H-AAO membranes due to its lower chemical resistance to phosphoric acid etching compared to the H-AAO layer. Thus, the pores of the resulting H-AAO membrane are fully opened at both sides. Afterwards, the membranes were coated with a protective SiO_2_ conformal layer of 2 nm in thickness, deposited by ALD at 150°C from aminopropyltriethoxysilane (100°C), water (RT), and ozone (RT) that were employed as precursors and oxidant agent, respectively [23,24]. The back side of the H-AAO templates was coated by means of sputtering and further electrodeposition of a continuous gold layer, which serves as a working electrode in the subsequent electrodeposition process of multisegments of Co-Ni alloy. Multisegmented Co_54_Ni_46_/Co_85_Ni_15_ nanowire arrays were electrochemically grown from a Watts-type bath containing 0.36 M CoSO_4_, 0.04 M CoCl_2_, 0.76 M NiSO_4_, 0.13 M NiCl_2_, and 0.73 M H_3_BO_3_. The pH of the electrolyte was adjusted to a value of 4 to 4.2 by adding 1 M NaOH. Electrodeposition processes were carried out at 35°C under potentiostatic conditions in a three-electrode electrochemical cell equipped with a Ag/AgCl reference electrode with a 3 M KCl, an insoluble Pt mesh counter electrode, and the gold-coated H-AAO template acting as the working electrode. The composition of each individual segment of the multisegmented Co_54_Ni_46_/Co_85_Ni_15_ nanowire arrays was tuned by adjusting the deposition potential in the range between −0.8 and −1.4 V versus the reference electrode. The duration of the potentiostatic deposition pulses was adjusted accordingly with the estimated deposition rate at each potential in order to obtain longitudinal segments of around 300 to 400 nm in length for each Co-Ni single segment. After the Co-Ni electrodeposition process, gold caps of about 2 μm in length were deposited in the upper part of the nanowires for protecting them from corrosion.

In order to perform a TEM characterization of individual multisegmented Co_54_Ni_46_/Co_85_Ni_15_ nanowires, it has been necessary to release them from the H-AAO template via chemical etching procedure. Firstly, the gold layer was partially removed by wet chemical etching in KI 0.6 M and I_2_ 0.1 M aqueous solution, and the SiO_2_ protective coating covering the empty parts of the H-AAO template was removed by dipping the sample in diluted HF. Afterwards, the alumina membrane, which contains embedded nanowire arrays, was immersed in a mixture of H_3_PO_4_ (6 wt.%) and CrO_3_ (1.8 wt.%) at 45°C for 48 h, resulting in the total dissolution of the alumina template. Free-standing nanowires, protected by a thin SiO_2_ coating layer and gold caps at both ends of the nanowires, were then filtered and suspended in absolute ethanol. Then, a small amount of nanowires was dispersed in ethanol-distilled water mixture (1:1). Subsequently, the obtained suspension was sonicated for 30 min at RT. Finally, a drop of the dispersed solution was placed in a *lacey carbon* grid and dried for 30 min, and afterwards, the solvent was evaporated in ambient environment. TEM studies were carried out in a *field emission gun* microscope FEI Titan 80–300 kV (Hillsboro, OR, USA), operated at 300 kV. Scanning transmission electron microscopy (STEM) and TEM modes have been used to obtain the micrographs. The STEM mode images have been registered using the high-angle annular dark-field (HAADF)-STEM detector. The HAADF detector collects electrons diffracted at high angles, which are chemically sensitive. In addition, local elemental analyses of cobalt and nickel content were carried out by STEM coupled to the EDS technique along the long and short axes of a single nanowire (EDS line scan) in order to gain information about the composition of each nanowire segments. The microstructure of such segments was investigated by SAED measurements.

Additionally, scanning electron microscope (JEOL 6610-LV, Akishima, Tokyo, Japan), equipped with EDS, was also employed for the morphological and compositional characterization of both the H-AAO templates and homogenous Co-Ni nanowires in order to determine the optimal synthesis conditions for the deposition of multisegmented Co-Ni nanowires.

The RT magnetic behavior of the multisegmented Co-Ni nanowire arrays was studied by means of vibrating sample magnetometer (VSM, Versalab-Quantum Design, San Diego, CA, USA) under a maximum applied magnetic field of ±30 kOe along both parallel and perpendicular directions with respect to the nanowire longitudinal axes.

## Results and discussion

**Figure 1:**
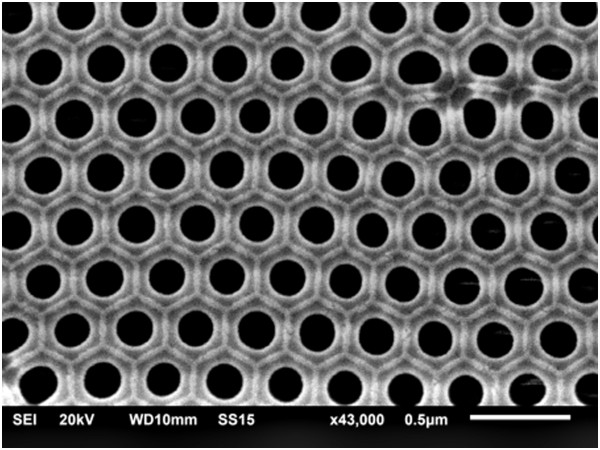
**SEM bottom view of a typical H-AAO membrane.** Employed for the electrochemical synthesis of the multisegmented Co-Ni nanowire arrays displaying the hexagonally ordered pore distribution with 180 ± 20 nm in size and pore interspacing of around 305 nm.

Figure 1 displays a SEM bottom view of the H-AAO membrane employed for the electrochemical synthesis of the multisegmented Co-Ni nanowire arrays, indicating the uniformity of the pore size (180 ± 20 nm) and pore interspacing (305 nm) of the highly ordered surface pore distribution with hexagonal symmetry achieved during the HA process.

**Figure 2:**
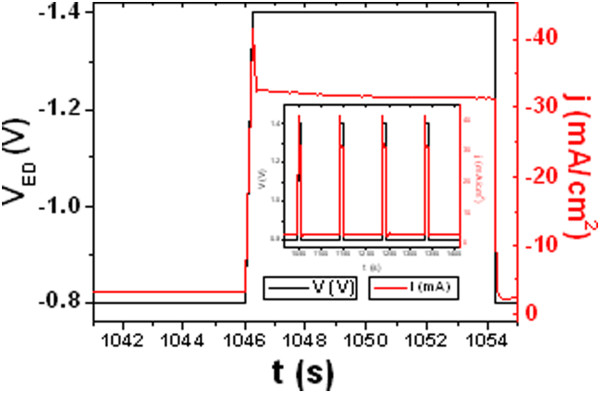
Pulsed electrodeposition potential sequence employed for the synthesis of multisegmented Co-Ni nanowires in H-AAO templates.

The pulsed electrodeposition potential sequence shown in Figure 2, employed for the synthesis of multisegmented Co-Ni nanowires, consisted of 25 cycles comprising a first deposition pulse of 86.83 s at −0.8 V followed by a second deposition pulse with a duration of 7.09 s at −1.4 V, which results in nanowires composed of 25 bi-segments consisting of Co_85_Ni_15_ and Co_54_Ni_46_ alloys having mean lengths of around 430 and 290 nm, respectively.

**Figure 3:**
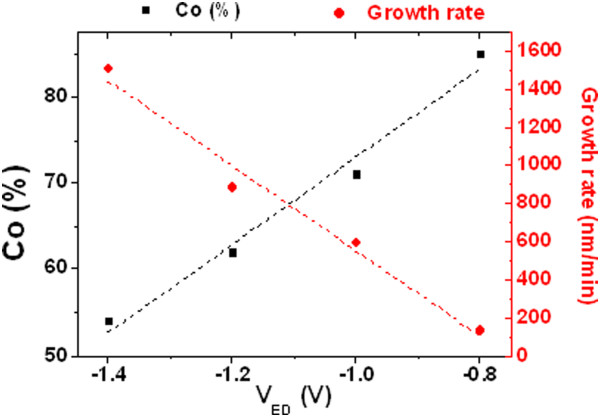
**Co content (left) and Co-Ni nanowire growth rate (right) dependence on the deposition potential, *V***_**ED**_**.**

The dependence of the composition and growth rate on the electrodeposition potential was determined by SEM and EDS studies of homogenous Co-Ni alloy nanowire arrays grown at several deposition potentials in order to fine-tune the parameters of the pulse sequence further employed for the fabrication of multisegmented Co_54_Ni_46_/Co_85_Ni_15_ nanowire arrays. These results are illustrated in Figure 3. The growth rate increases from 150 nm/min to 1,500 nm/min when the electrodeposition potential is decreased from −0.8 to −1.4 V, whereas the cobalt content of the nanowire alloy increases from 54 up to 85 at.% in the same voltage interval. The linear dependence on the electrodeposition potential exhibited by both the nanowire growth rate and Co content of the deposited alloys allows for a precise control on the composition and length of each individual segment during the electroplating of multisegmented Co_85_Ni_15_/Co_54_Ni_46_ alloy nanowire arrays.

**Figure 4:**
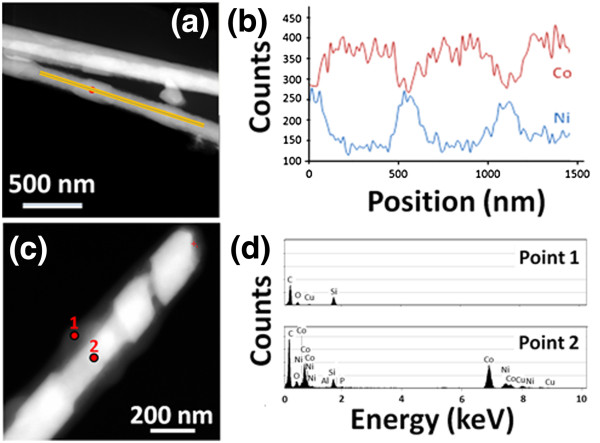
**STEM-HAADF images, variation of Co and Ni contents, and EDS analysis. (a**, **c)** STEM-HAADF images of multisegmented Co-Ni nanowires. **(b)** Variation of cobalt (red) and nickel (blue) contents along the orange line highlighted in (a) determined via elemental analysis by EDS line scan. **(d)** EDS analysis measured in the two points marked in the HAADF-STEM image of **(c)**. The presence of Si and O and the absence of Co and Ni can be seen in the EDS spectrum of point 1.

STEM-HAADF images of Co-Ni nanowires are shown in Figures 4a,c. These micrographs reveal that the nanowires present a core (bright)/shell (dark) structure together with a multisegmented core feature. The difference of contrast is due to the difference in the atomic number of the elements present in the metallic core and the SiO_2_ surface layer. In addition, analysis realized in different points of a single nanowire corroborated the core/shell structure of the nanowires (see Figure 4c,d). The EDS line scan performed in the middle along the longitudinal axis of a single Co_85_Ni_15_/Co_54_Ni_46_ segmented nanowire (Figure 4a,b) and also across the transversal direction (data not shown) discloses that the Co and Ni content distributions are very uniform in each segment of the nanowire. On the other hand, the EDS line scan along the single nanowire axis (Figure 4a,b) indicates that the distribution of both Co and Ni fluctuates among adjacent segments, and thus, the composition of segments alternates between Co_55_Ni_45_ and Co_82_Ni_18_, in agreement with previous results obtained from the SEM/EDS characterization of homogeneous Co-Ni alloy nanowires. Furthermore, the segments that appear as thinner and longer in Figure 4a,c are rich in Co, being the widest and shortest segments those with a higher Ni content. The difference in lengths found between core segments with different Co/Ni ratio can be attributed to deviations of their respective effective deposition rates from that shown in Figure 3. On the other hand, the diameter modulation of each Co-Ni segment could be an indication of a slight chemical etching of the surface of Co-rich segments during the process of releasing nanowires from the H-AAO template, which is however not observed in the Ni-richer segments, as a result of the different corrosion resistance behaviors of Co_85_Ni_15_ and Co_54_Ni_46_ alloys [25].

It is worth to point out that the composition profiles obtained from the linear EDS scans of Figure 4b performed in the multisegmented Co-Ni nanowires by STEM mode do not fit to pulse function as the applied deposition potentials do, probably ascribed to relaxation effects that occur during the deposition processes.

**Figure 5:**
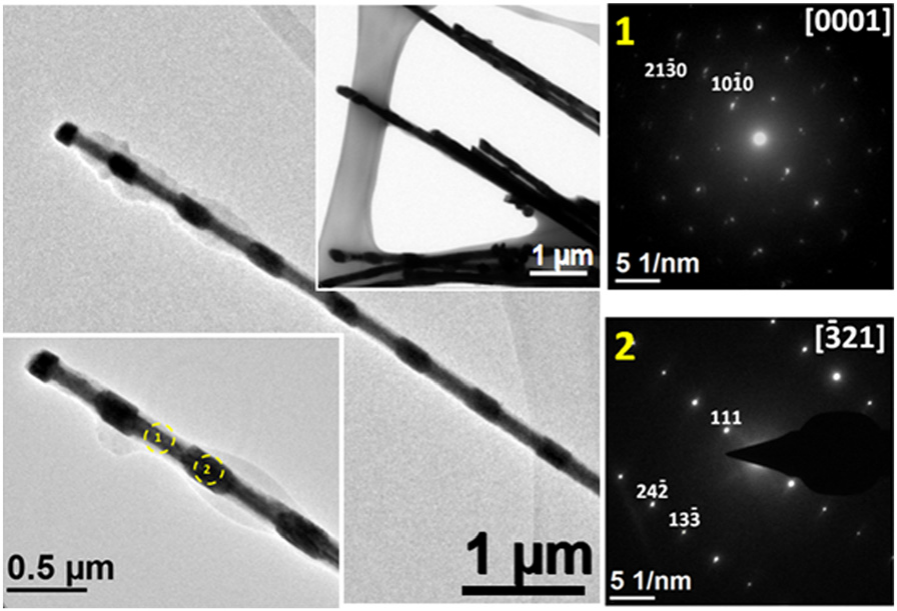
**TEM images and SAED patterns.** The left image shows TEM images of multisegmented Co-Ni nanowires. The right image shows SAED patterns of the different nanowire segments marked in the left image of the figure. SAED pattern with number (1) can be indexed to the [0001] zone axis of a Co-Ni alloy with a hcp structure. SAED pattern number (2) can be indexed to the [−321] zone axis of a Co-Ni alloy with a fcc structure.

The left image of Figure 5 shows typical TEM images of the Co-Ni nanowires, where their multisegmented structure is also clearly evidenced. The mean length of the Co_54_Ni_46_ alloy segments estimated from these images was 290 ± 30 nm, and the mean length of the segments with Co_85_Ni_15_ alloy composition was 422 ± 50 nm. Figure 5 also presents at the right image SAED patterns of two different representative segments of the same Co-Ni nanowire (highlighted by circles and numbers in the TEM micrograph), which allows to distinguish between the structure of both segments, being hcp for the Co_85_Ni_15_ segment (1), while fcc for the Co_54_Ni_46_ (2).

The local examination of the microstructure and composition of the different nanowire segments revealed that their crystalline structure changes as the Co/Ni ratio is modified. Particularly, it was found that nanowire segments containing at least 60% of cobalt display SAED patterns which correspond to hcp single crystals grown along the <10-10 > direction. On the other hand, nanowire segments containing below 60% of cobalt exhibit SAED patterns corresponding to a Co-Ni alloy single crystal with a fcc structure, where the <111 > direction lies along the nanowire axis. Representative examples are shown in Figure 5. In particular, the segment highlighted with number (1) on the left of Figure 5 has a composition of Co_83_Ni_17_, which was determined by EDS operating the microscope in TEM mode. The spots of the corresponding SAED pattern can be indexed to the [0001] zone axis of a Co-Ni single crystal with hcp structure. In addition, it is observed that the <10-10 > direction lies along the nanowire axis. On the other hand, the segment highlighted with number (2) having Co_52_Ni_48_ composition exhibits a SAED pattern that can be indexed to the [−321] zone axis of a Co-Ni alloy with fcc structure, where the <111 > direction lies along the nanowire axis. Interestingly, in several of these SAED patterns, the diffraction spots appear slightly elongated, or well, two or three spots appear very close. This fact evidences a texture that could be originated by fluctuations in the distribution of the Co/Ni ratio into the same segment and/or the effect of transversal stresses produced by the confined growth into the pores of the alumina template. The appearance of the hcp structure for Co-Ni alloys with high Co content is in agreement with its equilibrium phase diagram [26]. However, it is worth noting that in some of the studied nanowire segments, the concentration fluctuations and structural differences have also appeared, probably as a consequence of the non-equilibrium nature of the electrodeposition processes.

**Figure 6:**
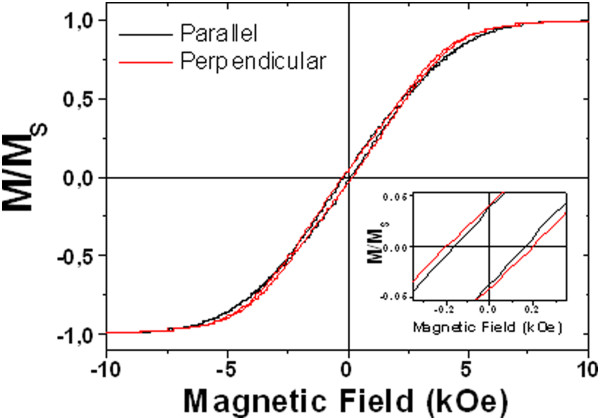
**Room temperature hysteresis loops of multisegmented Co**_**54**_**Ni**_**46**_**/Co**_**85**_**Ni**_**15 **_**nanowires.** Measured in the parallel and perpendicular directions with respect to the nanowire long axis. The inset shows an enlargement in the low-field region.

The RT hysteresis loops depicted in Figure 6 show small coercive field values of *H*_C_ = 150 and 194 Oe for the parallel and perpendicular directions, respectively. The reduced remanence (*m*_r_ = *M*_r_ / *M*_S_) in both directions takes similar values close to 0.04. These results point out that the array of multisegmented Co-Ni nanowires does not clearly show an easy magnetization axis, indicating that the longitudinal magnetic shape anisotropy of the multisegmented nanowire arrays is strongly competing against the magnetocrystalline anisotropy induced by the presence of hcp crystals with their easy axis lying in the perpendicular direction with respect to the long axis of the nanowires. Furthermore, dipolar interactions among adjacent barcode nanowires having narrow segments with different compositions and crystalline structures can have a strong effect in the resulting hysteresis loops, smearing the characteristic features of the abovementioned anisotropies.

## Conclusions

ALD SiO_2_-coated multisegmented Co_85_Ni_15_/Co_54_Ni_46_ nanowire arrays, with around 180 nm in diameter and made of tens of alternating segments with respective compositions of Co_54_Ni_46_ and Co_85_Ni_15_ having several hundreds of nanometers in length (around 290 nm for the segments of Co_54_Ni_46_ alloy and around 430 nm for the Co_85_Ni_15_ segments), have been synthesized by template-assisted electrochemical deposition into the pores of H-AAO templates by alternately varying between two different deposition potentials. Both Co content and nanowire growth rate vary quasi-linearly with the deposition potential. Based on this relation, the desired Co-Ni composition in each individual segment can be simply controlled by properly choosing the deposition potential. SAED allows distinguishing between the structures of both nanowire segments, being hcp for the Co_85_Ni_15_ segment, while fcc for the Co_54_Ni_46_ one, due to the influence of higher presence of fcc Ni in the alloy rather than changes induced during the electrodeposition dynamics. This technique allows not only for tuning the composition of the nanowires but also their crystalline structure in each different nanowire segments, which also affects the magnetic behavior making this system magnetically isotropic.

## Competing interests

The authors declare that they have no competing interests.

## Authors’ contributions

The experiments presented in this work were conceived and designed by VMP, KN, and CL. JG, LI, and VV prepared the samples during the laboratory tasks on the SiO_2_ atomic layer deposition on the alumina membranes. Co-Ni magnetic nanowires were microscopically characterized by JG, LI, VV, EDB-C, RM-R, AP, and CL, and they analyzed the SEM, TEM, STEM, and SAED results. JG, VV, and VMP carried out the magnetometry measurements on the samples and analyzed the results. JG, VV, RM-R, CL, DG, KN, and VMP analyzed and discussed the results obtained from the experiments. JG, VV, CL, and VMP wrote the manuscript, and the last version of this was revised by all the authors (VMP, JG, LI, VV, DG, KN, EDB-C, RM-R, AP, and CL). All authors read and approved the final manuscript.

## References

[B1] Arico AS Bruce P Scrosati B Tarascon J-M van Schalkwijk W Nanostructured materials for advanced energy conversion and storage devices Nature Mater 2005 8 366 377 15867920 10.1038/nmat1368

[B2] Rao CNR Deepak FL Gundiah G Govindaraj A Inorganic nanowires Progress in Solid State Chemistry 2003 8 5 147 10.1016/j.progsolidstchem.2003.08.001

[B3] Rao CNR Govindaraj A Synthesis of inorganic nanotubes Adv Mater 2009 8 4208 4233 10.1002/adma.200803720

[B4] Hangarter CM Lee Y-I Hernandez SC Y-h C Myung NV Nanopeapods by galvanic displacement reaction Angew Chem Int Ed 2010 8 7081 7085 10.1002/anie.201001559 20715230

[B5] Li X Wang Y Song G Peng Z Yu Y She X Li J Synthesis and growth mechanism of Ni nanotubes and nanowires Nanoscale Res Lett 2009 8 1015 1020 10.1007/s11671-009-9348-0 20596512 PMC2893774

[B6] Proenca MP Sousa CT Ventura J Vazquez M Araujo JP Distinguishing nanowire and nanotube formation by the deposition current transients Nanoscale Res Lett 2012 8 280 10.1186/1556-276X-7-280 22650765 PMC3583116

[B7] Masuda H Fukuda K Ordered metal nanohole arrays made by a two-step replication of honeycomb structures of anodic alumina Science 1995 8 1466 1468 10.1126/science.268.5216.1466 17843666

[B8] Nielsch K Müller F Li A-P Gösele U Uniform nickel deposition into ordered alumina pores by pulsed electrodeposition Adv Mater 2000 8 582 586 10.1002/(SICI)1521-4095(200004)12:8<582::AID-ADMA582>3.0.CO;2-3

[B9] Vázquez M Pirota K Hernández-Vélez M Prida VM Navas D Sanz R Batallán F Velázquez J Magnetic properties of densely packed arrays of Ni nanowires as a function of their diameter and lattice parameter J Appl Phys 2004 8 6642 10.1063/1.1687539

[B10] Vega V Böhnert T Martens S Waleczek M Montero-Moreno JM Görlitz D Prida VM Nielsch K Tuning the magnetic anisotropy of Co-Ni nanowires: comparison between single nanowires and nanowire arrays in hard-anodic aluminum oxide membranes Nanotechnology 2012 8 465709 10.1088/0957-4484/23/46/465709 23095457

[B11] Lee W Ji R Gösele U Nielsch K Fast fabrication of long-range ordered porous alumina membranes by hard anodization Nature Mater 2006 8 741 747 16921361 10.1038/nmat1717

[B12] Tang X-T Wang G-C Shima M Magnetic layer thickness dependence of magnetization reversal in electrodeposited CoNi/Cu multilayer nanowires J Magn Magn Mater 2007 8 188 196 10.1016/j.jmmm.2006.06.032

[B13] Shakya P Cox B Davis D Giant magnetoresistance and coercivity of electrodeposited multilayered FeCoNi/Cu and CrFeCoNi/Cu J Magn Magn Mater 2012 8 453 459 10.1016/j.jmmm.2011.08.023

[B14] Clime L Zhao SY Chen P Normandin F Roberge H Veres T The interaction field in arrays of ferromagnetic barcode nanowires Nanotechnology 2007 8 435709 10.1088/0957-4484/18/43/435709 17455508

[B15] Maijenburg AW George A Samal D Nijland M Besselink R Kuiper B Kleibeuker JE ten Elshof JE Electrodeposition of micropatterned Ni|Pt multilayers and segmented Ni|Pt|Ni nanowires Electrochim Acta 2012 8 123 128

[B16] Talapatra S Tang X Padi M Kim T Vajtai R Sastry GVS Shma M Deevi SC Ajayan PM Synthesis and characterization of cobalt–nickel alloy nanowires J Mater Sci 2009 8 2271 2275 10.1007/s10853-008-3015-1

[B17] Vivas LG Vázquez M Vega V García J Rosa WO del Real RP Prida VM Temperature dependent magnetization in Co-base nanowire arrays: role of crystalline anisotropy J Appl Phys 2012 8 07A325 10.1063/1.3676431

[B18] Vivas LG Vázquez M Escrig J Allende S Altbir D Leitao DC Araujo JP Magnetic anisotropy in CoNi nanowire arrays: analytical calculations and experiments Phys Rev B 2012 8 035439

[B19] Vega V Prida VM García JA Vázquez M Torque magnetometry analysis of magnetic anisotropy distribution in Ni nanowire arrays Physica Status Solidi A 2011 8 553 558 10.1002/pssa.201026390

[B20] Pirota KR Béron F Zanchet D Rocha TCR Navas D Torrejón J Vázquez M Knobel M Magnetic and structural properties of fcc/hcp bi-crystalline multilayer Co nanowire arrays prepared by controlled electroplating J Appl Phys 2011 8 083919 10.1063/1.3553865

[B21] Allende S Vargas NM Altbir D Vega V Görlitz D Nielsch K Magnetization reversal in multisegmented nanowires: parallel and serial reversal modes Appl Phys Lett 2012 8 122412 10.1063/1.4754117

[B22] Rheem Y Yoo B-Y Beyermann WP Myung NV Electro- and magneto-transport properties of a single CoNi nanowire Nanotechnology 2007 8 125204 10.1088/0957-4484/18/12/125204

[B23] Knez M Nielsch K Niinistö L Synthesis and surface engineering of complex nanostructures by atomic layer deposition Adv Mater 2007 8 3425 3438 10.1002/adma.200700079

[B24] Bachmann J Zierold R Chong YT Hauert R Sturm C Schmidt-Grund R Rheinländer B Grundmann M Gösele U Nielsch K A practical, self-catalytic, atomic layer deposition of silicon dioxide Angew Chem Int Ed 2008 8 6177 6179 10.1002/anie.200800245 18618880

[B25] Srivastava M Selvi VE Grips VKW Rajam KS Corrosion resistance and microstructure of electrodeposited nickel–cobalt alloy coatings Surf Coat Tech 2006 8 3051 3060 10.1016/j.surfcoat.2006.06.017

[B26] Hansen M Constitution of Binary Alloys 1958 2 New York: McGraw-Hill 486

